# Mapping Research Trends in One Health: A Comprehensive Review

**DOI:** 10.7759/cureus.70047

**Published:** 2024-09-23

**Authors:** Rini Raphael, Deepthi AS, Preetha Karnaver, R. Aruna Devy, Devi Priya M, Jobin Jose

**Affiliations:** 1 Department of Zoology, Carmel College (Autonomous), Mala, IND; 2 Department of Botany, Catholicate College, Pathanamthitta, IND; 3 Department of Zoology, Christian College, Chengannur, IND; 4 Department of Zoology, St. Thomas College, Ranni, IND; 5 Department of Botany, St. Thomas College, Ranni, IND; 6 Department of Library Science, Marian College Kuttikkanam (Autonomous), Kuttikkanam, IND

**Keywords:** bibliometric analysis, biblioshiny, one health, rstudio, vosviewer

## Abstract

The One Health approach, which emphasizes the interconnection between human, animal, plant, and environmental health, has gained significant attention as a framework for addressing global health challenges. This study presents a thorough bibliometric analysis of One Health research, utilizing the Web of Science Core Collection database, covering the period from 1989 to 2024. The analysis uncovers a substantial increase in scientific output over the years, reflecting the growing importance of this interdisciplinary field. The research reveals a notable shift in focus from traditional areas such as microbiology and infectious diseases to broader public health issues, including healthcare management, policy development, and food security, with particular attention to plant health, crop production, and the use of pesticides. Through advanced tools like Biblioshiny and VOSviewer, this study identifies prolific authors, leading journals, and emerging research themes shaping the One Health domain. The findings highlight the critical role of international collaboration in advancing this field and underscore the need for interdisciplinary approaches in both established and emerging research areas. By offering a comprehensive overview of the current landscape, this bibliometric evaluation serves as a valuable guide for researchers, policymakers, and stakeholders, emphasizing the essential role of One Health in developing strategies to combat complex health issues on a global scale.

## Introduction and background

In the past decade, much attention has been paid to the One Health concept as a means of comprehensively addressing global health challenges in a complex world. One Health underscores the interconnections between human, animal, and environmental health and promotes a multidisciplinary approach to issues such as zoonotic diseases, antimicrobial resistance (AMR), food safety, environmental pollution, and the effects of climate change on health systems [1].

The One Health approach emerged in response to the potential risk to human and animal health posed by an increased circulation of infectious agents, such as the global rise in zoonotic diseases [2]. One Health has been applied in diverse contexts, each with unique challenges and opportunities. For example, in India, the concept is still in its infancy and remains difficult to implement due to the lack of a legal framework, poor coordination among involved agencies, and insufficient surveillance mechanisms for animal diseases. On the other hand, in countries like the Netherlands, One Health has been successfully integrated into public health policy, with strong collaboration between human, animal, and environmental health sectors. The Dutch approach has effectively addressed zoonotic diseases, such as avian influenza, through coordinated surveillance, rapid response systems, and public health interventions [3].

The One Health approach has been adopted at various levels worldwide, evolving into a quadripartite collaboration among the Food and Agriculture Organization (FAO), the WHO, the World Organisation for Animal Health (WOAH), and the United Nations Environment Programme (UNEP). This alliance, particularly active in the Asia-Pacific region, has been instrumental in advancing interagency coordination on zoonotic diseases and AMR [4]. This approach has also been integrated into policies targeted at tackling AMR in Western Europe by increasing multidisciplinary research into zoonotic diseases [5]. Environmental health often contributes crucially but is very frequently the most overlooked part of the One Health triad. Dash S and Rath A have insisted on addressing environmental factors, which include pollution and ecosystem degradations, that act as carriers for infectious diseases and increase the threat of AMR [6]. One Health strategies need to integrate measures for environmental health if truly sustainable health outcomes are to be achieved.

This wide acceptance and success would be guaranteed by the inclusion of One Health principles not only in veterinary curricula, as recommended by the WOAH, but also across broader educational programs, including those for health professionals such as doctors, veterinarians, public health practitioners, and environmental scientists. Expanding One Health education to medical schools and other health-related fields, as well as incorporating its concepts at earlier educational stages, could further enhance interdisciplinary collaboration and health outcomes. Machalaba C et al. demonstrated that integrating One Health principles into medical education and clinical practice improves the efficient utilization of resources, resulting in better health outcomes at both local and global levels [7]. Moreover, increasing the approach and making it more inclusive and expansive, including diverse knowledge systems such as those of Indigenous peoples, has been identified as a prerequisite to the evolution and success of One Health [8]. The approach provides an integrated framework for responding to global health challenges through multi-sectoral collaboration. However, it must overcome significant hurdles related to resource constraints, lack of awareness, and poor coordination among all stakeholders [9-10]. If environmental health is further integrated into the One Health approach and educational efforts are expanded, then this will lay very firm ground for a global health strategy: that health systems shall be more sustainable and resilient.

Bibliometric analysis is one of the quantitative methods for identifying influences in research and tendencies by examining publication, citation, and collaboration patterns in networks [11-15]. Two important tools for performing tasks associated with this are VOSviewer and Biblioshiny for RStudio [16-17]. VOSviewer excels at generating and visualizing bibliometric networks such as co-authorship and citation networks; it aids researchers in discovering relations and trends within large datasets [18-21]. Biblioshiny is a web-based application of the Bibliometrix package in RStudio, easy to use, and simplifies the analysis of trends in publications and citation patterns without programming skills [22-26]. In combination, these tools allow for the realization of full analysis, intuitively visualized, of scientific literature regarding influential works and emerging research areas.

The objectives of the paper include delving into details of the 'One Health' concept and exploring why the approach represents an integrative solution that links health for humans, animals, and the environmental ecology. This level of challenge is further epitomized in the need for interdisciplinary collaboration in addressing complex health challenges, zoonotic diseases, food insecurity, and climate change. Furthermore, this paper aims to promote a full-scale bibliometric assessment of 'One Health' research by intellectually mapping the key contributors in the field and its emerging trends. This integrated study maps the intellectual structure of One Health for research on a subject domain that lacks prior mapping, shedding light on the gaps and future directions for researchers and policymakers to address complex health problems spanning the human, animal, and environmental domains.

## Review

Materials and methods

The Web of Science Core Collection was chosen as the primary bibliographic data source for this study due to its comprehensive coverage of high-quality journals, making it a preferred option over other databases [27-28]. The publications were retrieved using the keyword "One Health," without imposing any language restrictions, and focusing exclusively on journal articles. A total of 6,259 documents were collected from 1,441 different sources, spanning the period from 1989 to 2024. In the second phase of data curation, we excluded Reviews, Conference papers, Book Chapters, Editorials, Books, Letters, Notes, and Short Surveys, narrowing the dataset to include only articles. The resulting dataset was saved in both CSV and RIS formats, and bibliometric analysis was conducted using VOSviewer and Biblioshiny software to extract meaningful insights from the data.

The investigation summarized in Table 1 provides key insights into a comprehensive dataset spanning from 1989 to 2024. It includes 1,441 sources, such as journals and books, encompassing a total of 6,259 documents. The data shows a substantial annual growth rate of 16.68%, indicating a rapidly expanding body of research. The documents have an average age of 4.83 years and receive an average of 14.73 citations each, reflecting their impact and relevance in their respective fields. Collectively, these documents cite 221,907 references. In terms of document contents, there are 9,643 unique Keywords Plus entries and 12,538 unique author keywords, demonstrating a diverse range of topics and research areas covered in the dataset. The dataset features contributions from 32,844 authors, with 302 authors having single-authored documents, highlighting a mix of individual and collaborative research efforts. Notably, 329 documents are single-authored, and on average, each document involves 7.22 co-authors, signifying a high level of collaboration. Additionally, 40.39% of the documents involve international co-authorship, underscoring the global nature of the research. Regarding document types, the dataset primarily consists of 6,172 articles, with an additional 87 early access articles, indicating the predominance of traditional research articles while also including cutting-edge early access publications.

**Table 1:** Key aspects of investigation.

Description	Results
Timespan	1989-2024
Sources (Journals, Books, etc)	1441
Documents	6259
Annual Growth Rate %	16.68
Document Average Age	4.83
Average citations per doc	14.73
References	221907
Document Contents	
Keywords Plus (ID)	9643
Author's Keywords (DE)	12538
Authors	
Authors	32844
Authors of single-authored docs	302
Authors Collaboration	
Single-authored docs	329
Co-Authors per Doc	7.22
International co-authorships %	2528 (40.39%)
Document Types	
Article	6172
Article; early access	87

Annual scientific production

**Figure 1:**
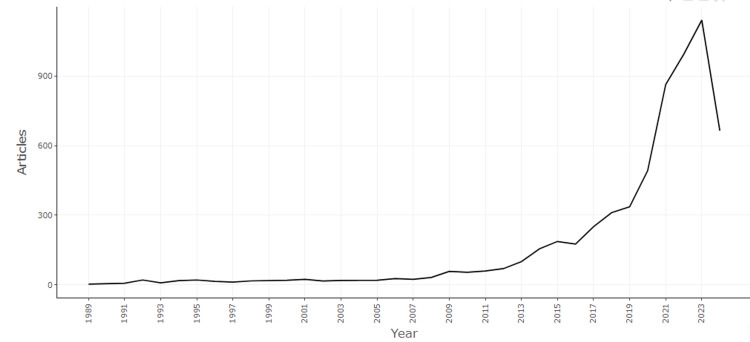
Annual scientific production.

Figure 1 illustrates the annual scientific production in "One Health" research from 1989 to 2024, revealing a clear trend of increasing research activity over the years. Initially, from 1989 to 2000, the number of articles published each year remained relatively low, ranging from 3 to 24 articles annually. A notable increase begins in the early 2000s, with the number of articles gradually rising, reaching 32 by 2008, and continuing to grow steadily each year. A significant surge in publications starts from 2009, with the number of articles jumping to 58 and accelerating in subsequent years. The period from 2012 to 2019 shows consistent growth, with 336 articles published by 2019. The most dramatic growth occurs from 2020 onwards, with 492 articles in 2020, 863 in 2021, 993 in 2022, and a peak of 1,140 articles in 2023. For 2024, up until June, 664 articles have already been published, indicating that the year's total is likely to exceed previous years if the trend continues. Overall, this figure highlights a significant and accelerating increase in "One Health" research output, reflecting growing recognition and interest in the interconnectedness of human, animal, plant, and environmental health.

Most relevant authors

Table 2 highlights the most prolific authors in "One Health" research, showcasing their contributions by the number of articles published. Leading the list is Zinsstag J, with 44 articles, indicating significant impact and dedication to the field. Following are Lincopan N with 31 articles, and Biondo AW with 27 articles. Behravesh CB has authored 25 articles, while Cardoso B has contributed 23 articles. Degeling C, Esposito F, and Zhang Y each have 21 articles to their names, reflecting their active participation in the research community. Finally, Hasler B and Kmetiuk LB have each published 20 articles, rounding out the top contributors. This table underscores the contributions of these key researchers to the advancement and dissemination of "One Health" knowledge.

**Table 2:** Most relevant authors.

Authors	Articles
Zinsstag J	44
Lincopan N	31
Biondo AW	27
Behravesh CB	25
Cardoso B	23
Degeling C	21
Esposito F	21
Zhang Y	21
Hasler B	20
Kmetiuk LB	20

Most relevant sources

Table 3 presents the most important sources and highlights key journals and publications that significantly contribute to the field based on the number of articles published. Leading the list with 320 articles, One Health is the most prolific source, reflecting its central focus on the interdisciplinary "One Health" approach. Antibiotics-Basel, with 180 articles, emphasizes research on antibiotics, AMR, and related topics, crucial aspects of "One Health." Frontiers in Veterinary Science, publishing 166 articles, underscores the importance of veterinary science in the "One Health" paradigm. Known for its broad scientific scope, PLoS ONE has contributed 133 articles, highlighting its role in disseminating diverse research related to "One Health." Animals, with 130 articles, focuses on animal health and welfare, integral components of the "One Health" approach. Frontiers in Public Health, publishing 118 articles, reflects the public health dimension of "One Health," covering disease prevention, health policy, and community health. Frontiers in Microbiology, with 116 articles, highlights the microbial aspects of "One Health," including studies on pathogens, microbiomes, and infectious diseases. Revue Scientifique Et Technique-Office International Des Epizooties, contributing 114 articles, is key for veterinary and epidemiological research on zoonotic diseases and global animal health. Microorganisms, publishing 105 articles, focuses on microbiological research relevant to "One Health," particularly in understanding pathogen-host interactions. Pathogens, with 104 articles, emphasizes research on various pathogens, their epidemiology, and control measures, crucial for "One Health" strategies. These sources collectively underscore the interdisciplinary nature of "One Health" research, covering topics from veterinary science and microbiology to public health and antibiotic resistance, playing a vital role in advancing knowledge and fostering collaboration across different scientific domains to address complex health challenges.

**Table 3:** Most relevant sources.

Sources	Articles
One Health	320
Antibiotics-Basel	180
Frontiers in Veterinary Science	166
PLoS ONE	133
Animals	130
Frontiers in Public Health	118
Frontiers in Microbiology	116
Revue Scientifique et Technique-Office International des Epizooties	114
Microorganisms	105
Pathogens	104

Most locally cited sources

Table 4 provides an overview of the most locally cited sources in "One Health" research, highlighting the journals and publications with the highest impact in this field based on the number of articles cited. Leading the list is PLoS ONE with 5,731 articles, indicating its significant influence and contribution to the dissemination of "One Health" research. Following this are Emerging Infectious Diseases with 4,365 articles, reflecting its critical role in addressing infectious disease issues. PLoS Neglected Tropical Diseases is also highly cited with 3,195 articles, emphasizing its focus on tropical diseases within the "One Health" framework. Frontiers in Microbiology (3,089 articles) and Journal of Antimicrobial Chemotherapy (2,895 articles) are key sources, indicating their relevance in microbiology and antimicrobial research. Journal of Clinical Microbiology follows closely with 2,774 articles, underscoring its importance in clinical research. The Lancet (2,606 articles) is a prominent source, reflecting its broad impact on health research. Antimicrobial Agents and Chemotherapy with 2,419 articles, and Applied and Environmental Microbiology with 2,252 articles, highlight the importance of antimicrobial and environmental microbiology studies. Finally, Science of the Total Environment with 2,216 articles indicates the significance of environmental science in the "One Health" approach. This table underscores the diverse range of journals that contribute to the field, reflecting the interdisciplinary nature of "One Health" research, spanning infectious diseases, microbiology, clinical studies, antimicrobial research, and environmental science.

**Table 4:** Most locally cited sources.

Sources	Citations
PLOS ONE	5731
Emerg Infect Dis	4365
PLOS Neglect Trop D	3195
Front Microbiol	3089
J Antimicrob Chemother	2895
J Clin Microbiol	2774
Lancet	2606
Antimicrob Agents Ch	2419
Appl Environ Microb	2252
Sci Total Environ	2216

Countries' scientific productions

Table 5 highlights the scientific production of various countries in the field of "One Health" research by the number of articles published. The USA leads significantly with 5,198 articles, indicating its dominant role and substantial contribution to the field. The UK follows with 2,256 articles, demonstrating strong research activity. Brazil is also a major contributor with 1,679 articles, reflecting its active involvement in "One Health" research. Australia has produced 1,429 articles, showing its significant participation as well. China (1,236 articles) and Italy (1,235 articles) have almost equal contributions, indicating their strong research presence. Canada (1,187 articles), France (1,092 articles), Germany (1,071 articles), and Spain (950 articles) also show notable contributions, underscoring the global engagement in "One Health" research across diverse regions. This table illustrates the widespread international commitment to advancing knowledge and addressing health issues through a "One Health" approach.

**Table 5:** Countries' scientific productions.

Region	Articles
USA	5,198
UK	2,256
Brazil	1,679
Australia	1,429
China	1,236
Italy	1,235
Canada	1,187
France	1,092
Germany	1,071
Spain	950

Trend topics

**Figure 2:**
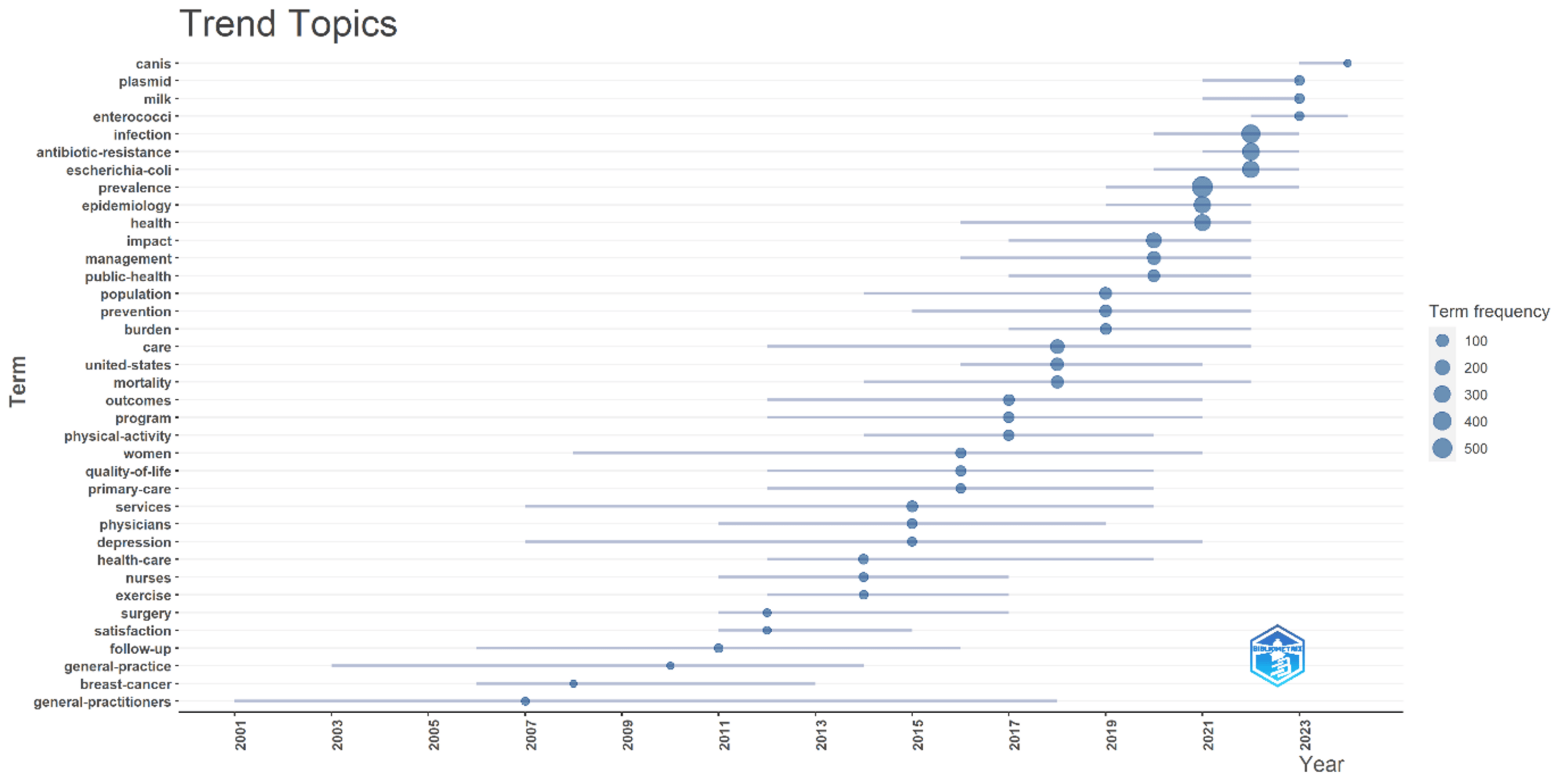
Trend topics.

The trend topics graph in Figure 2 illustrates the evolution and frequency of key terms in "One Health" research from 2001 to 2024, with larger circles representing higher term usage. From the early 2000s to the late 2010s, terms like "canis," "plasmid," "milk," "enterococci," "infection," and "antibiotic-resistance" emerged and increased in frequency, reflecting a growing interest in microbiology, infectious diseases, and antibiotic resistance. From the mid-2010s onwards, terms such as "epidemiology," "health," "impact," "management," "public-health," and "prevention" became prominent, indicating a shift towards broader public health concerns, disease management, and preventive measures. In the late 2010s to early 2020s, there was an increased focus on terms like "burden," "care," "mortality," "outcomes," "program," and "population," showing an emphasis on healthcare delivery, health outcomes, and population health. In recent years (2020s), terms such as "physical-activity," "quality-of-life," "primary-care," "services," "physicians," "depression," "health-care," "nurses," "exercise," "surgery," "satisfaction," "follow-up," "general-practice," "breast-cancer," and "general-practitioners" have gained prominence, highlighting a broadening scope to include mental health, healthcare services, quality of life, and specific medical conditions. This trend analysis underscores the dynamic and evolving nature of "One Health" research, reflecting changes in focus areas over time, from microbiology and infectious diseases to broader public health, healthcare management, and quality of life issues, suggesting a more integrated approach that addresses both biological and systemic aspects of health.

Thematic map

**Figure 3:**
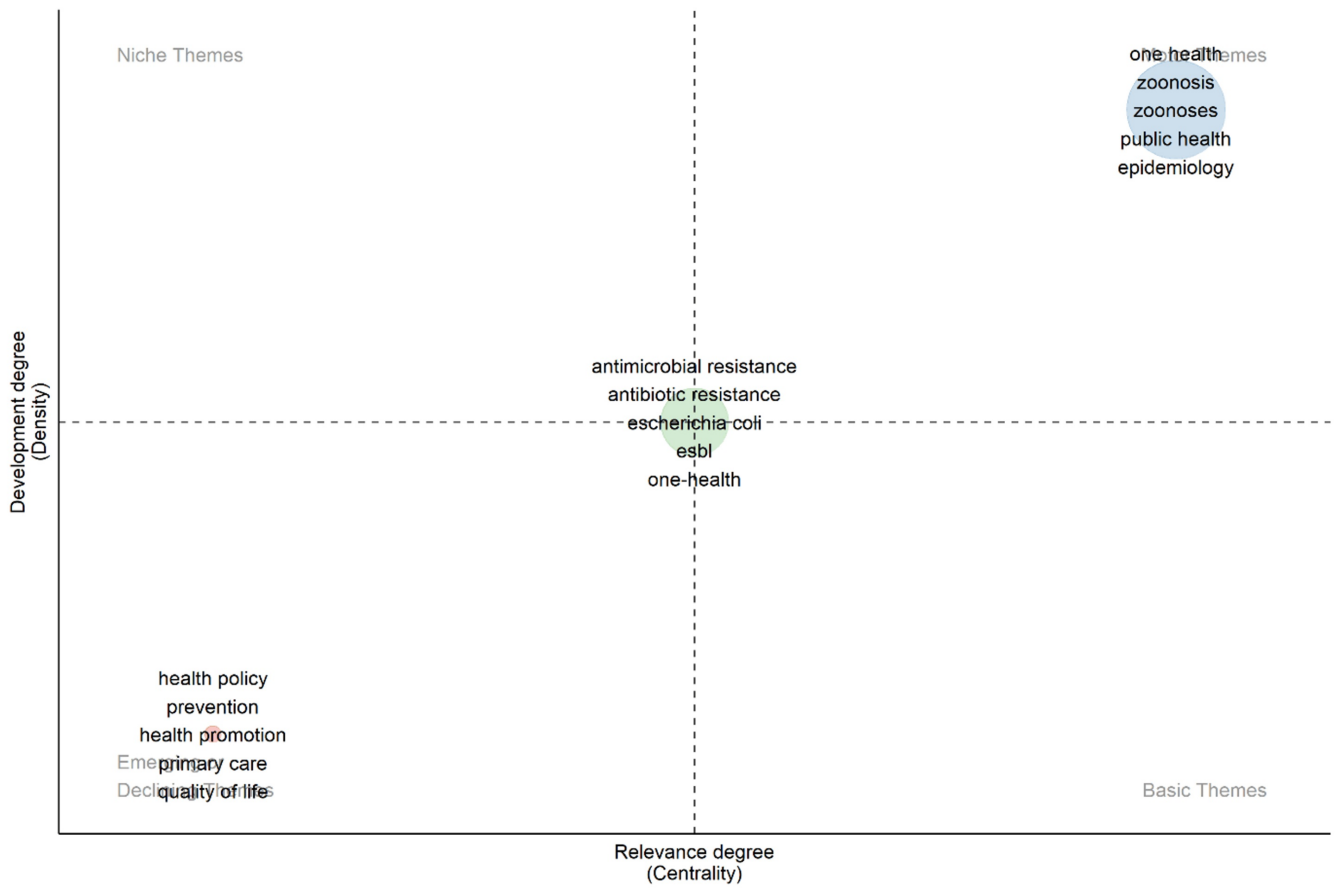
Thematic map.

The thematic map in Figure 3 visualizes the development and relevance of key themes in "One Health" research, dividing them into four quadrants based on the degree of development (density) and relevance (centrality). The upper-right quadrant (Motor Themes) contains highly developed and relevant themes such as "one health," "zoonosis," "zoonoses," "public health," and "epidemiology," indicating their central role in the field. The upper-left quadrant (Niche Themes) is empty, suggesting no themes are highly developed but of low relevance. The lower-left quadrant (Emerging or Declining Themes) includes less developed and less relevant themes like "health policy," "prevention," "health promotion," "primary care," and "quality of life," which might be emerging or declining in focus. The lower-right quadrant (Basic Themes) contains central but not yet well-developed themes such as "antimicrobial resistance," "antibiotic resistance," "Escherichia coli," "Extended-Spectrum Beta-Lactamases (ESBL)," and "one health," highlighting foundational areas of active exploration. Overall, the map underscores the strategic importance of both well-established and emerging themes in shaping the future of "One Health" research.

Topic dendrogram

**Figure 4:**
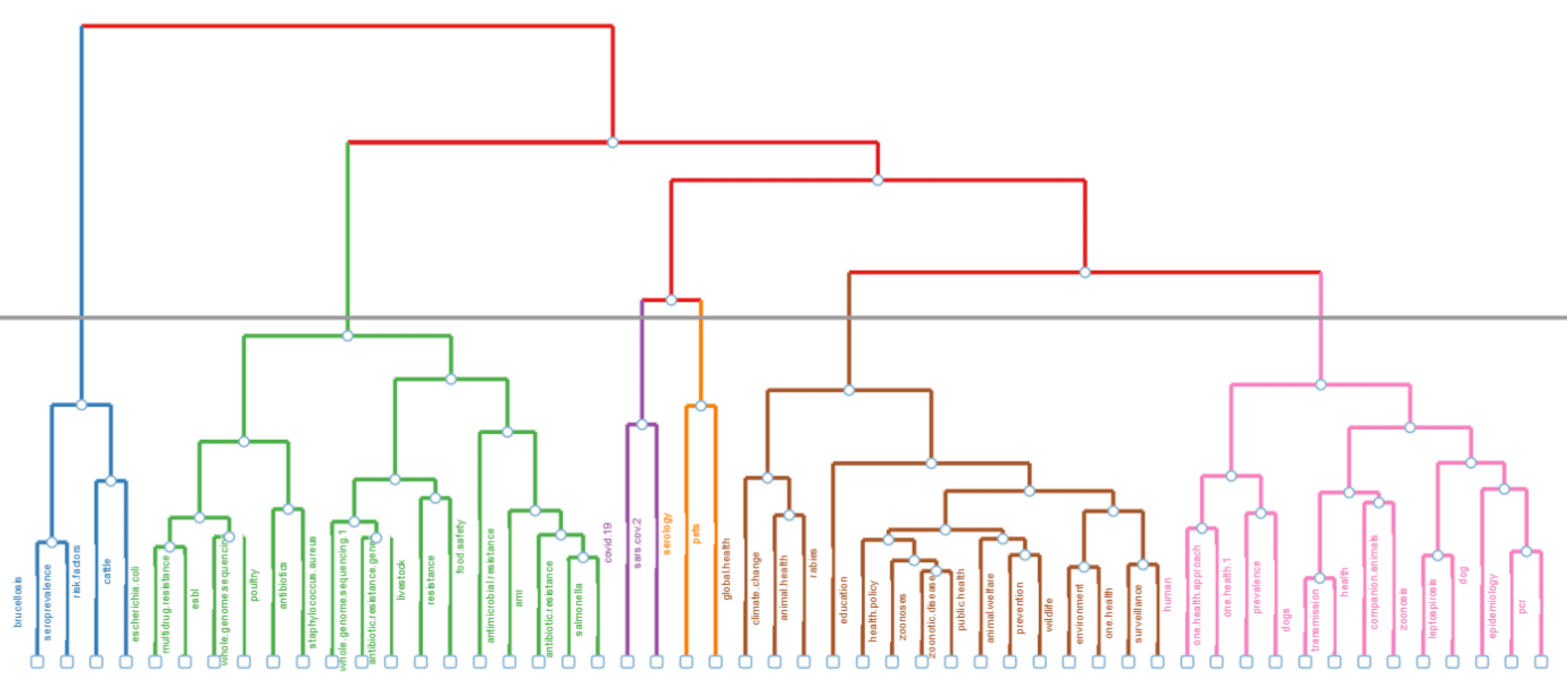
Topic dendrogram.

The topic dendrogram in Figure 4 for "One Health" research visualizes the hierarchical clustering of various topics within the field, showing how closely related different areas of study are. The dendrogram can be interpreted through its high-level clusters. Cluster One (Blue) includes terms such as "brucellosis," "seroprevalence," "leptospirosis," "Escherichia coli," and "lactococcus," indicating a focus on specific bacterial infections and their prevalence, highlighting a microbiological and epidemiological aspect within "One Health." Cluster Two (Green) involves terms like "whole genome sequencing," "antibiotic resistance," "Staphylococcus aureus," "livestock," "food safety," and "AMR," reflecting a broader focus on genomic research, antibiotic resistance, and food safety, with significant attention to livestock and public health implications. Cluster Three (Red) includes terms such as "serology," "SARS-CoV-2," "pets," and "COVID-19," pointing to research on viral diseases, zoonotic transmission, and the role of pets in disease spread, emphasizing the importance of recent pandemic studies and serological testing.

Cluster Four (Orange) includes terms such as "global health," "climate change," "animal health," and "education," suggesting a focus on the broader impact of global health issues, environmental changes, and educational initiatives within the "One Health" framework. Cluster Five (Brown) contains terms like "zoonoses," "public health," "animal welfare," "prevention," and "surveillance," indicating a focus on zoonotic diseases, public health strategies, animal welfare, and the importance of surveillance and prevention methods in "One Health." Cluster Six (Pink) includes terms such as "prevalence," "dogs," "transmission," "companion animals," "epidemiology," and "polymerase chain reaction (PCR)," highlighting research on disease prevalence, transmission dynamics, companion animals, and molecular epidemiology techniques like PCR.

The dendrogram reveals a strong emphasis on health-related topics within "One Health" research, including microbiology, public health, disease prevention, genomic studies, and environmental health. The clustering indicates that "One Health" research covers a wide array of interconnected topics, emphasizing the integration of human, animal, and environmental health. Cluster One focuses on bacterial infections and their epidemiology, Cluster Two highlights genomic research and antibiotic resistance, and Cluster Three emphasizes viral diseases and zoonotic transmission. Cluster Four addresses global health and environmental changes, while Cluster Five concentrates on zoonotic diseases, public health, and animal welfare. Finally, Cluster Six delves into disease prevalence, transmission, and molecular epidemiology. This visualization underscores the interdisciplinary nature of "One Health" research, pointing to the need for collaborative and holistic strategies to address health challenges across different domains.

Co-occurrence of all keywords

**Figure 5:**
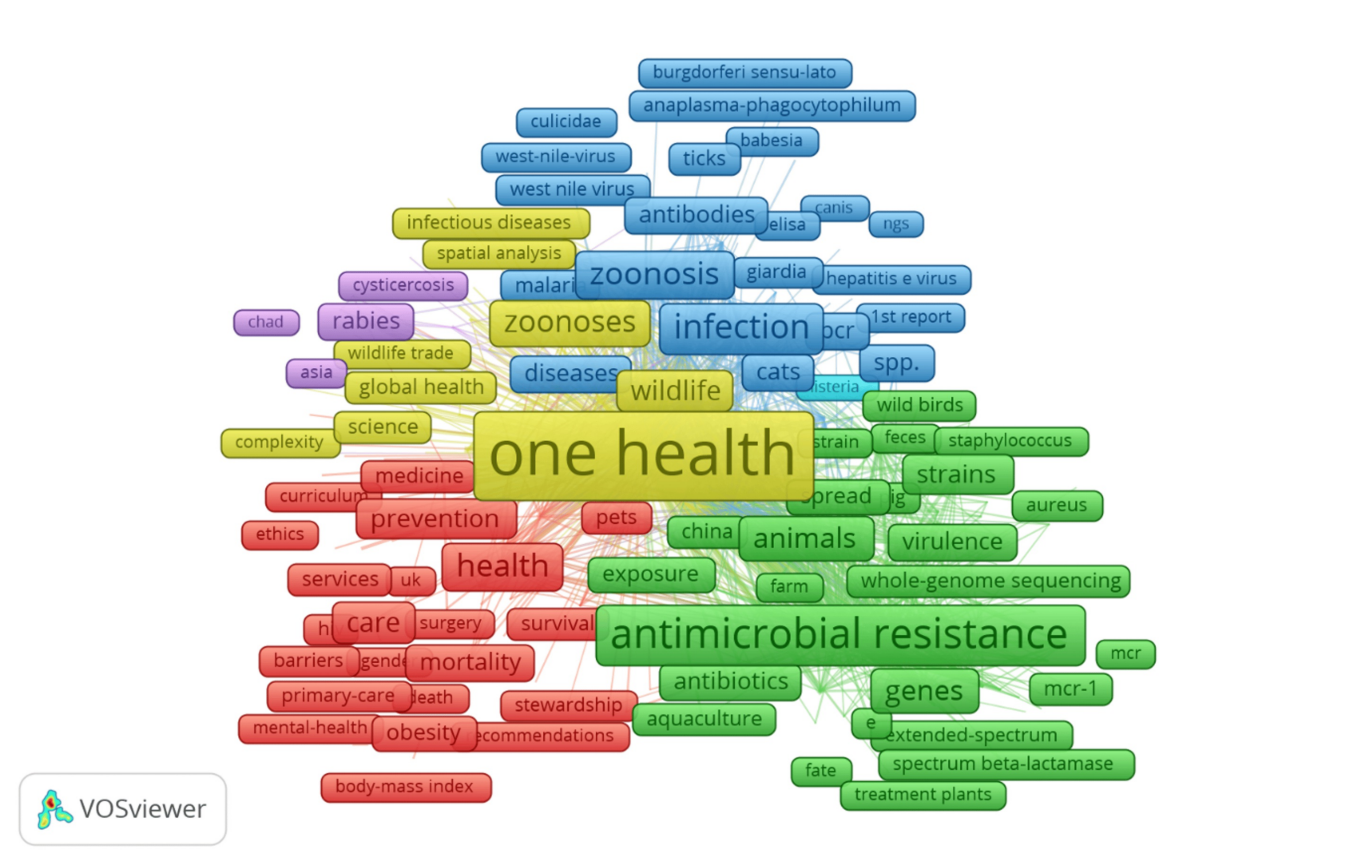
Co-occurrence of all keywords.

Figure 5 presents a co-occurrence network of keywords within "One Health" research, visualizing the interrelation of terms with a minimum occurrence threshold of 10. The network includes 872 keywords out of a total of 20,084, categorized into seven distinct clusters, each representing different thematic areas within the field. Cluster 1 (Red) comprises 255 keywords related to health systems, prevention, health behavior, and themes like "care," "mortality," "risk," and "education." Cluster 2 (Green), with 231 keywords, focuses on AMR and related topics such as "antibiotics," "identification," and "whole-genome sequencing." Cluster 3 (Blue) contains 196 keywords emphasizing infectious diseases and zoonoses, featuring terms like "zoonosis," "infection," and "SARS-CoV-2." Cluster 4 (Yellow), with 130 keywords, centers around animal health and zoonoses, including "rabies" and "vaccination." Cluster 5 (Purple) with 56 keywords includes terms related to tropical diseases like "malaria." Clusters 6 (Cyan) and 7 (Orange) are smaller, representing specialized or emerging areas. This network underscores the interdisciplinary nature of "One Health" research, highlighting major focus areas and the interconnectivity of various themes, reflecting the comprehensive approach required in this field.

Co-authorship of countries

**Figure 6:**
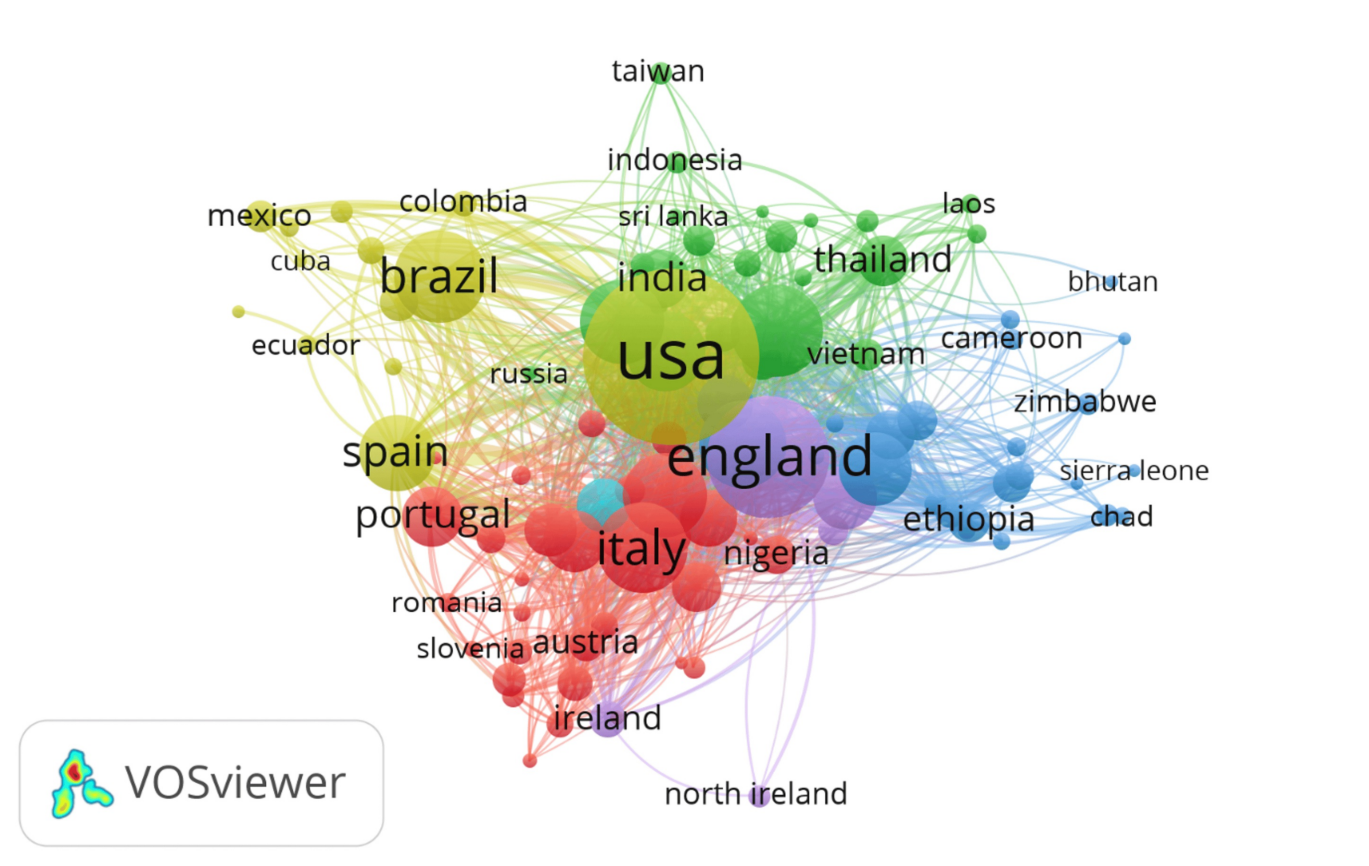
Co-authorship of countries.

Figure 6 depicts the network visualization of co-authorship among countries in the "One Health" research field, with a minimum threshold of 10 documents per country. Out of 173 countries, 101 meet this threshold, resulting in a network of eight distinct clusters. Cluster 1 (Red) includes 33 countries such as England, Portugal, and Italy, indicating strong collaborative networks within Europe and connections to other regions. Cluster 2 (Green), with 24 countries, features major contributors like the USA, India, and Thailand, highlighting extensive collaboration across North America and Asia. Cluster 3 (Blue), also consisting of 24 countries, includes significant players like Brazil and Ethiopia, representing collaborations primarily within South America and Africa. Cluster 4 (Yellow) comprises 14 countries, including Spain and Mexico, indicating a focus on collaboration within the Spanish-speaking world and Latin America. Cluster 5 (Purple) includes five countries such as Slovenia and Austria, representing specific regional collaborations. Cluster 6 (Cyan) contains only one country, likely representing a unique or emerging research area with limited but significant collaboration. The network visualization highlights the extensive and intricate collaboration patterns in "One Health" research across the globe, with major countries like the USA, England, Brazil, and India serving as central nodes, underscoring the importance of international cooperation in addressing complex health issues that span human, animal, and environmental health domains.

Discussion

The "One Health" approach, as reflected in the bibliometric analysis, underscores the increasing recognition of the interconnectedness between human, animal, and environmental health. The increase in publication output over recent years shows that globally, there is an increasing awareness of the need for interdisciplinary collaboration on health challenges that transcend traditional boundaries. The thematic evolution underway in "One Health" research, from an infectious disease and antibiotic resistance focus to broader public health concerns and environmental sustainability, demonstrates the dynamic nature of the field. At the same time, some topics' highest dominance, the case of zoonotic diseases and AMR, confirms their ability to remain relevant for the global health agenda. This also shows the strong contribution from countries like the USA, the UK, and Brazil, thus highlighting high international collaboration, which has been considered a very important ingredient in tackling the complexities and global nature of health issues.

Research gaps and future directions

Although there has been considerable growth in 'One Health' research, several significant gaps remain. Beyond zoonotic diseases and AMR, critical areas such as the impact of climate change on emerging diseases and the health of humans, animals, and plants are underexplored. Additionally, the role of behavior and belief systems in shaping environmental health and disease transmission patterns needs further investigation. The influence of production systems on the economic and health outcomes of societies is another area requiring more research attention. Moreover, there is a growing need to develop robust frameworks for evaluating health intervention tools within the scope of the One Health approach. Addressing these gaps will provide a more comprehensive understanding of the interconnected health challenges facing our world.

Practical implications

The findings from this bibliometric analysis have several practical implications for policymakers, researchers, and practitioners in the 'One Health' field. Increasing research trends underscore the rationale for continued investments in specific 'One Health' initiatives, particularly those aimed at fostering interdisciplinarity in zoonotic disease control, AMR management, and climate change-related health interventions. Policymakers at national and international levels need to consider integrating "One Health" principles into national and international health strategies in areas like disease surveillance, environmental protection, and biosecurity. For researchers, the analysis highlights the importance of expanding research into underexplored areas like environmental health and the effects of climate change. For practitioners, especially those involved in public health, veterinary medicine, and environmental science, these findings provide insights into how to work toward more holistic conceptualizations of health that actually get to the roots of health problems and not just their symptoms. Further, the global dimension of "One Health" research necessitates international collaboration and sharing of knowledge to decisively deal with health challenges across national borders.

## Conclusions

The bibliometric analysis of "One Health" research conducted in this study underlines how fast-paced and interdisciplinary this field is. It has shown that, with steep growth in scientific output and international collaboration, "One Health" has expanded to encompass a wide array of health-related issues, ranging from infectious diseases and antimicrobial resistance to broader public health, healthcare management, and environmental sustainability. Such an analysis identifies contributors, key journals, and emerging trends, consolidating knowledge about the intellectual structure and development of the field. The results emphasize that only through continued inter- and transdisciplinary dialogue and collaboration, including broad-ranging areas of research inquiry, can such complex and interrelated global health challenges begin to be addressed. As "One Health" increasingly transcends its niche, it offers a transformative model for global health that addresses resilience and sustainability in light of new and emerging health threats.
